# Successfully treating 90 patients with obsessive compulsive disorder in eight days: the Bergen 4-day treatment

**DOI:** 10.1186/s12888-018-1887-4

**Published:** 2018-10-04

**Authors:** Gerd Kvale, Bjarne Hansen, Thröstur Björgvinsson, Tore Børtveit, Kristen Hagen, Svein Haseth, Unn Beate Kristensen, Gunvor Launes, Kerry J. Ressler, Stian Solem, Arne Strand, Odile A. van den Heuvel, Lars-Göran Öst

**Affiliations:** 1 Haukeland University Hospital, OCD-team, 5021 Bergen, Norway; 2 Department of Clinical Psychology, University of Bergen, Bergen, Norway; 3 McLean Hospital, Belmont, MA USA; 4 Harvard Medical School, Boston, MA USA; 5 Molde Hospital, Molde, Norway; 6 Nidaros DPS, Division of Psychiatry, St. Olav University Hospital, Trondheim, Norway; 7 Oslo University Hospital, Oslo, Norway; 8 Sørlandet Sykehus, Kristiansand, Norway; 9 Department of Psychology, Norwegian University of Science and Technology, Trondheim, Norway; 10 Norwegian OCD-foundation, Ananke, Oslo, Norway; 11 Department of Psychiatry and Department of Anatomy & Neurosciences, VU university medical center (VUmc), Amsterdam, The Netherlands; 12 Department of Psychology, Stockholm University, Stockholm, Sweden

**Keywords:** Obsessive-compulsive disorder, OCD, 4-day treatment, Exposure

## Abstract

**Background:**

Oslo University Hospital, Norway, had by autumn 2016, accumulated a waiting list of 101 patients with obsessive-compulsive disorder (OCD) who had a legal right to receive treatment by a specialized OCD team. In this challenging situation, the Bergen OCD-team suggested to solve the problem by offering all patients an option for the rapid Bergen 4-day treatment (B4DT). The B4DT is an individual treatment delivered during four consecutive days in a group of six patients with the same number of therapists. The approach has previously shown a post-treatment response rate of 90% and a 3-month remission rate of 70%.

**Methods:**

Ninety-seven of the wait-list patients were available for the scheduled time slots, and 90 received the 4-day format during 8 days (45 patients each week). The therapists were recruited from 22 different specialized OCD-teams from all over Norway, and 44 (68%) had not previously delivered the 4-day format.

**Results:**

Post-treatment; 91.1% of the patients were classified as responders, and 72.2% were in remission. At 3-month follow-up; 84.4 were classified as responders and the remission rate was 67.7%. Oslo University Hospital now offers the 4-day treatment as standard treatment for OCD.

**Conclusions:**

We conclude that the B4DT is an acceptable and potentially effective OCD-treatment.

## Background

In Norway, all patients with obsessive-compulsive disorder (OCD) are granted empirically supported treatment by a specialized OCD-team [1], and 30 teams have been established since 2012. At Oslo University Hospital (OUH) the consequence of this new rule of granted treatment was a tripling of patients from 2014 to 2016, and more than 100 patients were waiting for treatment by the end of 2016. The most probable reason is that many more OCD-patients than previously applied for treatment when they realized they might be granted empirically supported treatment. While OUH employed a protocol for individual exposure and response prevention (ERP) [2] where patients typically are treated over 12–16 weeks, the OCD-team at Haukeland University Hospital, Bergen, has developed a novel treatment format where ERP is delivered during just four consecutive days. Despite the relatively brief treatment, the Bergen 4-day treatment (B4DT) has been shown to yield a good outcome [3–5]. At post-treatment assessment the proportion of responders varied from 83% [4] to 93.8% [5] with a weighted mean of 89.4%. At 6- or 12-month follow-up the mean response rate was 82.4%. The post-treatment remission rate varied between 73.8% [4] and 77.1% [3] with a weighted mean of 76.0%, and at follow-up the average rate was 69.7%. Also, the 4-day format has shown to be acceptable for the patients and has a 'low drop-out rate; 0.7% (only 1 out of 142 patients; [3–5]. However, these studies have had small sample sizes (*N* = 35, 42, and 65) so larger trials are warranted.

There are a few published studies on concentrated ERP for OCD (using Yale-Brown Obsessive Compulsive Scale as primary outcome measure) but no randomized trial comparing it to weekly sessions of ERP. Franklin et al. [6] and Abramowitz et al. [7] published uncontrolled effectiveness studies on concentrated ERP using 15 2 hour sessions over 4 weeks and 12 sessions of 90 min over 4 weeks, respectively. Hiss et al. [8] and Lindsey et al. [9] published efficacy studies using 19 sessions of 90 min over 4 weeks and 15 2 hour sessions over 3 weeks, respectively. Finally, there are two non-randomized comparison trials involving concentrated ERP. Abramowitz et al. [10] compared 15 2 hour sessions over 3 weeks with 15 sessions over 8 weeks (twice weekly) and found that the concentrated treatment yielded a significantly higher proportion of recovered patients post-treatment but not at 3-month follow-up. Storch et al. [11] compared 14 sessions of 90 min over 3 weeks with 14 weekly sessions and found no significant differences at post- or at 3-month follow-up assessment. Combining the Y-BOCS data for these six studies (*N* = 266) yielded a weighted mean at pre-treatment of 26.2 (SD 4.9) and at post-treatment of 11.9 (6.9), with a within-group effect size (Cohen’s *d*) of 2.42. This can be compared to 2.06 for standard ERP in the meta-analysis by Öst et al. [12]. Thus, previous versions of concentrated ERP have used daily sessions of 90 or 120 min over 3–4 weeks, which is quite different to the B4DT, consisting of four sessions of 3–8 h across four consecutive days. Since longer versions of concentrated ERP seem to yield somewhat better effects than standard ERP it is possible that our highly concentrated version yields even better effects.

The leader of the Bergen OCD-team (GK) was made aware of the treatment delay problem at OUH and suggested offering the 4-day treatment to patients on the waiting list, which OUH accepted. The 4-day treatment is best described as “individual treatment in a group setting” since the ratio between therapist and patients is 1:1 in groups which usually range from three to six patients. In order to treat 100 patients, eight parallel groups during two separate weeks would be needed. It was decided to offer OCD-therapists from the other specialized OCD-teams in Norway the opportunity to participate, and by doing so to start their training in the 4-day format. This logistically demanding project of treating 100 OCD-patients in 8 days was initiated during the spring of 2017. The present paper reports on the results.

Based on the outcomes of three previous effectiveness studies from our team [3–5] we predicted that the outcome of the present study would be equally good since the disorder and treatment were the same. Also, because the three previous studies [3–5] on the B4DT were all carried out with, to a large extent new therapists, we expected that the B4DT would be effective also with a substantial number of new therapists. Thus, the present study can be seen as an example of systematic replication [13] having the purpose to test the outcome when a large number of OCD-patients are treated at a new clinic by new therapists.

## Methods

### Participants and procedure

**Fig. 1:**
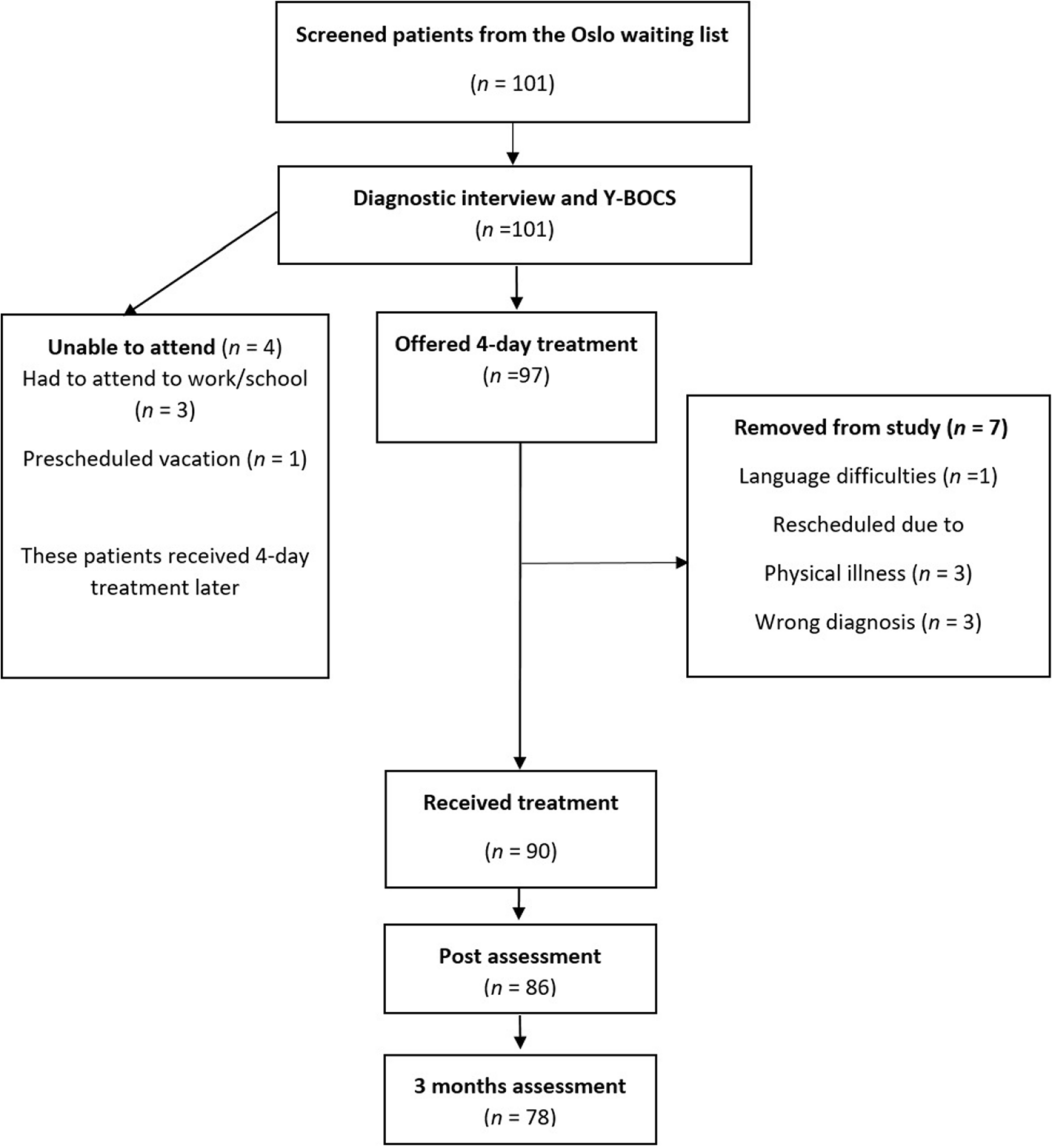
Flow chart of study participants

In Norway, OCD-patients with a principal DSM-5 diagnosis of OCD [14] are entitled to empirically supported treatment from an outpatient OCD-team. In February 2017, the 101 patients on the OUH waitlist were offered the 4-day treatment. Inclusion criteria were a diagnosis of OCD and patients had to be fluent in Norwegian. Exclusion criteria included suicidality, psychosis, and active substance abuse. Patients declining would receive standard care at OUH. Four were unable to attend due to school obligations, work, and a prescheduled vacation and 97 patients were scheduled (see flow chart in Fig. 1).

Patients referred to OUH had all been diagnosed with OCD (by a psychiatrist or clinical psychologist at the OUH OCD-team) and severity of OCD had been assessed with a Y-BOCS interview before being placed on the waiting list. Diagnoses were assessed using the MINI International Neuropsychiatric Interview [15]. For the present study, patients on the waiting list were formally referred to the OCD-team in Bergen, and they were re-assessed before starting treatment by using the OCD entry questions from DSM-5 as well as another Y-BOCS interview. This re-assessment was necessary as some patients had been waiting for treatment for many months. When formally referred to Bergen, patients were asked to complete a number of questionnaires administrated online (see measures) prior to the treatment, and to complete these questionnaires post-treatment as well as at 3-month follow-up.

Y-BOCS at post-treatment, as well as at 3-month follow-up, were conducted over the phone by specially trained clinical psychologists who worked at other clinics and were not involved in the treatment. At 3-month follow-up, 20% of the sample was randomly selected to be re-interviewed within 1 week by another independent psychologist. The inter-rater reliability, Intra Class Coefficient (3.1) = 0.93, was excellent.

The treatment was conducted in Oslo by bringing in therapists from all over Norway. Three of the 97 patients became ill (two with flu, one with a minor bleeding during pregnancy). One patient turned out to have severe language problems, and three were misdiagnosed (one had OCD-like symptoms which turned out to be due to a neurological disorder; one was preparing for a transsexual operation and the OCD-like symptoms were related to this, and one patient had a primary diagnosis of generalized anxiety disorder). Thus, the number of OCD patients who received the 4-day treatment was 90. All of these patients completed treatment so the attrition rate was 0%.

#### Demographics

Mean age was 32.7 (*SD =* 9.7), 57% were women, and 58% single. A total of 78% were either working or studying, whereas 22% received different social benefits. Mean years of education was 12.6 (*SD =* 3.5). Thirty-two patients reported to have family members with OCD (see Table 1 for details).

**Table 1:** Demographic and diagnostic characteristics of the sample (*N* = 90)

Demographics	*n* (%)/ M (SD)	Comorbidity	
Age	32.70 (9.72)	GAD	27 (30.0%)
Years of education	12.60 (3.46)	Depression	20 (22.2%)
Female gender	51 (56.7%)	Bipolar	1 (1.1%)
Single	52 (57.8%)	Panic	14 (15.5%)
Working	51 (56.7%)	Tourette	2 (2.2%)
Studying	19 (21.1%)	Specific phobia	4 (4.4%)
Social benefits	20 (22.2%)	Social phobia	11 (12.2%)
Previous treatment	79 (87.8%)	BDD	1 (1.1%)
Psychotropic meds	40 (44.4%)	Health anxiety	2 (2.2%)
GAF-S (0–100)	51.73 (5.50)	Anorexia	1 (1.1%)
GAF-F (0–100)	57.54 (9.96)	Bulimia	2 (2.2%)
Age at OCD onset	15.47 (6.55)	ADHD	1 (1.1%)
Years with OCD	16.12 (10.40)		

*Note: GAF* general assessment of functioning, *GAD* generalized anxiety disorder, *BDD* body dysmorphic disorder, *ADHD* attention deficit hyperactivity disorder

#### OCD severity and comorbidity

Mean pre-treatment score on the Yale-Brown Obsessive Compulsive Scale (Y-BOCS) was 26.16 (*SD =* 3.37), 77% had severe OCD (Y-BOCS score of 24–38), and 23% had a moderate OCD (Y-BOCS score of 18–23). A total of 86.7% had received previous treatment trials, but none of the patients had received CBT for OCD with including ERP procedures. Patients with previous therapy trials (*M* = 26.25, *SD* = 3.47) did not differ significantly from treatment naïve (*M* = 25.45, *SD* = 2.62), *t*(88) = 0.73, *p* = .47. A total of 56 patients (62%) had one or more comorbid disorders (see Table 1 for details).

#### Pharmacological treatment

Forty patients used psychotropic medications: 35 used antidepressants, one antiepileptic medication, eight antipsychotics, four stimulants, five anxiolytics, three hypnotics, and one received drug assisted treatment for substance abuse. Patients with and without psychotropic medication did not differ on Y-BOCS scores, *t*(88) = .014, *p* = .99, GAD-7 scores *t*(88) = .41 *p* = .69, or PHQ-9 scores, *t*(88) = 1.10, *p* = .27, at pre-treatment.

No changes were applied to medication, but the patients were informed that the use of anxiolytics was prohibited during and after the 4-day treatment. Patients were not receiving other treatment during the treatment period.

#### Preparing the patients for treatment

In order to ensure standardized information, patients received written information and watched a video presenting the outline of the 4-day treatment https://www.youtube.com/watch?v=nqx8knpy3i4 as well as a 6 minute description of the treatment made in 2014 by and broadcasted on Norwegian national TV (NRK) https://www.youtube.com/watch?v=ZRSExyZ3GPg. After accepting the treatment offer, they watched the following video describing the 4-day treatment in more detail https://www.youtube.com/watch?v=1Fnxt0_ljpY&t=1s.

The patients’ expectations of treatment outcome and evaluation of the treatment credibility, were assessed with an adapted version of the Borkovec and Nau [16] *Reaction to treatment scale* (0–100%). A score below 100% on any of the four questions was taken as an opportunity to clarify possible misunderstandings. The patients were instructed to suggest exposure tasks, and as guidance they were told that “exposures that their OCD would appreciate the least” often were the most relevant. During the week before treatment, the group leader (see below) called each patient and performed a standard interview, focusing on clarifying possible misunderstandings and ensuring that the patients understood that they were required to prepare exposure tasks.

#### Staffing the groups

In order to be able to treat 100 patients, two time slots were selected with eight groups of six patients in each. Therapists from 22 different OCD-teams in Norway wanted to participate, as well as a Scandinavian-speaking American colleague and three Icelandic psychologists. Therapists with the most extensive OCD-experience were given priority, as were therapists from teams that would be able to participate in both time-slots. When possible, the groups were staffed with 50% of qualified 4-day therapists, or therapists with at least one prior experience with the 4-day treatment. In order to qualify as a 4-day therapist he or she must have participated in a minimum of two 4-day groups and demonstrated competency in the exposure procedure evaluated independently by two 4-day experts. All group leaders had acted as therapists in minimum of six groups. In the Oslo case, group leaders did not have responsibility for a given patient, but rather was responsible to ensure that all therapists and patients received the necessary supervision, intervention, and assistance. In each group, one of the therapists also acted as “second in command”. Furthermore, each group had a person taking care of the logistics. In total, 66 therapists (including group leader) participated, 61 of them twice, and 44 of them without prior experience with the 4-day format.

### Preparing the therapists for the 4-day treatment and logistic

Groups were scheduled from Tuesday to Friday, and therapists met the day before for 8 hours in preparation, including introduction to the psychoeducation and a detailed outline of the 4-day treatment, given by the developers, Bjarne Hansen (BH) and Gerd Kvale (GK). Prior to this, all therapists received the same introduction to the 4-day treatment as the patients. The therapists also received all relevant information regarding their patients. GK and BH had daily meetings with each group leader and “second in command” to ensure that potential challenges were dealt with in accordance with the 4-day protocol.

Groups were located at different places at the OUH, minimizing external disturbances related to the number of parallel groups. Also, a centrally located “control center” led by GK and BH and staffed with support personnel was established.

### Treatment

The first of the 4 days (approximately 3 h) was allocated to psychoeducation and to prepare individual exposure tasks. The two middle days were dedicated to individually tailored and therapist assisted exposure training (8–10 h each day) in a wide range of OCD-relevant settings.

The main feature of the 4-day treatment is to teach the patients to actively approach whatever elicits the relevant anxiety or discomfort, and to help them systematically use the anxiety and discomfort as a cue to “**LE**an into **T**he anxiety” (LET-technique) instead of employing obvious or subtle avoidance. Typically, the therapist serves as a coach in the beginning, gradually leaving more responsibility to the patients. The therapists work as a team, which means that the patients are not necessarily working with the same therapist during the 2 days of exposure (see below). Rather, the team leaders allocate and reallocate therapists (during frequent and brief team-meetings) to ensure that the patients who are struggling are assisted by the most experienced therapists.

The exposures were interspaced with brief group meetings where each participant reported on how they were doing, especially on how they were practicing the LET-technique. The patients had individual exposure tasks for the afternoon and evenings, and reported progression back to the therapist with the last contact typically being at 9 p.m. Relatives and friends were invited to a psycho-educative meeting (one for each group) in the afternoon of day 3. The last day “lessons learnt” were summarized and plans for the next 3 weeks of self-administered exposures were made. The patients were informed how to contact the health care provider if an emergency situation should occur. The next 3 weeks, the patients were encouraged to report online every day on how they were practicing the LET-technique. The clinicians read the reports, without being in contact with the patients.

Three months after treatment, patients were invited to an individual session (30 min, conducted by the Oslo team) where their experiences in the period following treatment were discussed, and the principles of the LET-technique repeated. No exposure work was conducted in this session.

### Measures

*Yale-Brown Obsessive Compulsive Scale, Y-BOCS*; [17, 18] a semi-structured clinical interview that consists of 10 items (each 0–4) covering the severity of both obsessions and compulsions, and is a standard approach to assess OCD severity. The psychometric properties of the interview are well established. Y-BOCS has been found to be sensitive to change after treatment (e.g., 8).

*Patient Health Questionnaire-9, PHQ-9;* [19] is a frequently used self-administered screening instrument consisting of nine questions each rated on a 0–3 scale. A score of 10 or more is indicative of a depressive disorder [20]. The psychometric properties of the instrument are sound [20].

*Generalized Anxiety Disorder scale, GAD-7;* [20] is a self-administered screening instrument for symptoms of generalized anxiety consisting of seven items each rated on a 0–3 scale. The psychometric properties for the instrument are good [20].

### Benchmarking

As is customary in effectiveness studies we compared the mean Y-BOCS score and remission rate of the present sample and the average for published effectiveness studies on ERP. In order to do a fair comparison, we selected studies that had short-term follow-up assessment (3–6 months post-treatment) as in the present study, and used any format of ERP. The following studies were included in the benchmarking analysis [21, 22, 23–25] comprising a total of 381 patients fulfilling DSM-IV criteria for OCD.

### Statistical analyses

Statistical analyses were performed with SPSS version 24.0. Repeated measures ANOVA for Y-BOCS was conducted using pre-treatment, post-treatment, and follow-up scores. Effect sizes were calculated with Cohen’s *d*, defined as (Mpre – Mpost)/SDpre.

Treatment response was calculated based on the international consensus criteria [26] which requires a ≥ 35% reduction of the individual patient’s pre-treatment Y-BOCS score in order to be classified as a *clinically relevant response*. A patient is classified as *remitted* if the post-treatment Y-BOCS score is ≤12 points. For Y-BOCS scores there were no missing data at pre-treatment, whereas data were missing for four patients at post-treatment and 12 at follow-up. For PHQ-9 one case was missing at pre-treatment and 10 at post-treatment. For GAD-7 there were 2 patients with missing data at pre-treatment and 10 at post-treatment. Missing data were replaced using the expectation-maximization method of SPSS, version 24. The method was chosen to allow for repeated measures ANOVA. All data presented are an integrated part of the 4-day standard quality control procedure.

## Results

### Primary measures

Table 2 displays the results for Y-BOCS at pre-treatment, post-treatment, and 3-month follow-up. Repeated measures ANOVA (Wilks’ Lambda) found a significant effect of time, *F*(2) = 428.94, *p* < .001, partial Eta squared = .91. Mauchly’s test of sphericity was not significant (*p* = .263). A large effect size was observed with a Cohen’s *d* of 4.6 both at post-treatment and at follow-up. There were no significant changes in symptoms from post-treatment to follow-up assessment (*p* = .82).

**Table 2:** Means, standard deviations and effect sizes (Cohen’s *d*) for symptoms of OCD, anxiety, and depression

	M	SD	*d*
Y-BOCS			
Pre	26.16	3.37	
Post	10.54	4.61	4.63
3 months	10.68	6.31	4.59
GAD-7			
Pre	11.88	4.88	
Post	8.80	4.49	0.63
PHQ-9			
Pre	11.75	5.36	
Post	8.34	4.59	0.64

*Note: N* = 90. *d* = (Mpre – Mpost)/SDpre

### Clinically significant change in OCD-severity

At post-treatment, 91.1% of the patients had responded (≥35% improvement) and 72.2% were in remission (≥35% reduction and Y-BOCS score of ≤12). At 3-month follow-up, 84.4% had responded, and 67.8% were in remission.

Table 3 shows the clinical improvement at post-treatment and 3-month follow-up for the individual patients. Of the 65 patients who were in remission at post-treatment, 51 patients (78.5%) were classified as in remission at follow-up. Of the 17 patients who were classified as responders at post-treatment, 6 had become remitted at follow-up, 9 remained as a responder whereas 2 had deteriorated to the category of no change. Of the 8 patients who were classified as unchanged at post-treatment, 4 were in remission at follow-up, and 4 remained unchanged.

**Table 3:** Comparison of clinical improvement rates at post-treatment and follow-up

	3 months follow-up			
	Remission	Responded	Unchanged	Total
Post				
Remission	51	6	8	65
Responded	6	9	2	17
Unchanged	4	0	4	8
Total	61	15	14	90

Clinically significant change was also calculated for the two severity subgroups. For the moderate subgroup, 76.2% were classified as remitted at post-treatment and 23.8% showed no change. At follow-up 81.0% were remitted, 4.8% responded, and 14.3% showed no change. For the severe/extreme subgroup; 71.0% were remitted at post-treatment while 24.6% responded, and 4.3% showed no change. At follow-up 63.8% were remitted, 20.3% responded, and 15.9% showed no change.

Remission rates at post-treatment were not significantly different between the two severity subgroups, χ^2^ (1) = 0.22, *p* = .64. The same was true at follow-up, χ^2^ (1) = 2.18, *p* = .14.

### Depression and generalized anxiety

There were also significant decreases in symptoms of depression and generalized anxiety from pre- to post-treatment, equal to moderate effect sizes of .64 and .63 respectively (see Table 4).

**Table 4:** Severity of anxiety and depression at pre-treatment, post-treatment, and 3-months follow-up

	GAD-7		PHQ-9	
Severity	Pre	Post	Pre	Post
None	5 (5.6%)	13 (14.4%)	6 (6.7%)	15 (16.7%)
Mild	22 (24.4%)	43 (47.8%)	25 (27.8%)	42 (46.7%)
Moderate	37 (41.1%)	24 (26.7%)	32 (35.6%)	25 (27.8%)
Severe	26 (28.9%)	10 (11.1%)	27 (30.0%)	8 (8.9%)

*Note: Pre* Pre-treatment, *Post* Post-treatment. Cut-offs used for both GAD-7 and PHQ-9 were 0–4 (none), 5–9 (mild), 10–14 (moderate), 15 and above (severe)

### Comparison with our previous studies

Table 5 shows a comparison of the Y-BOCS data between the present study and our previous three studies [3–5] carried out in Bergen. The present sample of OCD-patients starts at the same severity level as the previous samples and has very similar post-treatment and 3-month follow-up results. There are no significant differences on Y-BOCS between the present and the previous samples at any time point.

**Table 5:** Comparison the 4-day treatment studies on Y-BOCS scores (*M* and *SD*)

Study				
Time point	Havnen (2014)	Havnen (2017)	Hansen (2018)	Present study
*N*:	35	42	65	90
Pre	26.1 (4.3)	25.7 (4.3)	25.8 (4.7)	26.2 (3.4)
Post	9.0 (4.8)	10.8 (3.9)	10.2 (5.1)	10.5 (4.6)
3 months	10.6 (7.0)	─	10.5 (5.9)	10.7 (6.3)
6 months	10.3 (5.7)	12.2 (6.4)	─	─
1 year	─	─	10.6 (7.0)	─
4 years	9.9 (7.4)			

*Note*: The 4-year time point pertain to the combination of the Havnen et al. 2014 and 2017 studies

### Benchmarking

Table 6 shows the comparison between the present sample and standard ERP from published uncontrolled effectiveness studies. The Oslo patients, treated with the Bergen 4-day treatment, had a significantly higher mean Y-BOCS score at pre-treatment. However, both at post-treatment and at 3-month follow-up their means were significantly lower than the average for the published effectiveness studies. The remission rate was also significantly higher for the Oslo patients than for the patients in the effectiveness studies.

**Table 6:** Comparison between the Bergen 4-day treatment and standard ERP in effectiveness studies

	Bergen 4-day		Effectiveness		Statistic
	*N*	*M* (*SD*)	*N*	*M* (*SD*)	*t-*test (*p*)
Y-BOCS					
Pre	90	26.2 (3.4)	381	24.4 (5.4)	3.02 (=.0026)
Post	90	10.5 (4.6)	374	18.2 (7.0)	9.93 (<.0001)
3 months	90	10.7 (6.3)	317	16.2 (8.1)	5.95 (<.0001)
Remission					Fisher’s test
Post	90	72.2%	110	35.5%	*p* < .0001
3 months	90	67.8%	110	45.5%	*p* = .0017

*Note*: Data for effectiveness studies were taken from uncontrolled studies of ERP having 3–6 month follow-up. N indicates the total number of participants across these studies. Remission was calculated as ≥35% reduction of pre-treatment Y-BOCS scores and ≤ 12 on post and follow-up Y-BOCS

## Discussion

The joint effort to erase the waiting list of more than 100 OCD-patients at Oslo University Hospital during 8 days worked well. In comparison, during the entire year of 2016 OUH was able to treat 70 patients. Ninety-one percent of the 90 patients treated with the 4-day format responded at post-treatment, and 68% were in remission at three-month follow-up.

The results from Oslo are basically the same as reported on the 4-day format in our previous effectiveness studies [3–5]. Furthermore, we have documented that the changes are maintained at 1-year follow-up [5] and at 4-year follow-up [27]. Compared to standard ERP treatment approaches, the 4-day format has a number of potential advantages. It has a low declining rate and attrition rate. Moreover, this treatment may have a significantly larger response rate and remission rate at post-treatment compared to standard ERP in effectiveness studies.

In the present study we demonstrated that we were able to upscale, and to help 90 moderately to severely affected OCD-patients in 8 days; 45 in each 4-day slot. This was done as a part of the public mental health care, with no selection of patients.

The 4-day format is often labelled “individual treatment in a group setting” indicating that the ratio between therapists and patients is 1:1. Basically, this means that each therapist is able to treat one patient in less than a week, which probably is highly cost-effective. All the participating therapists were experienced in treating patients with OCD using CBT, but 67% of them had previously not worked with the 4-day format and the clear majority of the therapists had never worked together before. Since each group is staffed with as many therapists as patients, the format serves as a good opportunity to work side-by-side with, and observe experienced 4-day therapists, and to get hands-on supervision. While model learning with hands-on supervision is an obvious approach for physicians who are going to learn surgery or any other medical discipline, it is rare in the mental health care. It is a unique experience to be able to observe six patients with OCD going through major changes in only 4 days. After the Oslo experience, all teams involved have asked to be fully trained in order to deliver the 4-day format.

There are different limitations of the current study. Evaluations of this treatment format has so far only been tested using open trial designs, which do not compare the effect of treatment with other established treatments or placebo control. A RCT is necessary to draw definitive conclusions about the efficacy and specificity of the 4-day treatment. Also, participants had not previously received evidence based treatment for OCD. This could limit generalizability of the findings. However, the present study was aimed at ordinary OCD-patients, not those who are treatment resistant. The study design and study population could be possible explanations for the good results. Another possible explanation is that the current concentrated format is different from that used in previous studies of concentrated treatment. They have used daily sessions of 90–120 min across 3–4 weeks, while we used 4 days and 3–8 h sessions. Longer sessions in highly concentrated ERP could yield better effects than standard ERP. Another issue concerns missing data as 13% of participants did not provide data at follow-up.

The present study tentatively indicates that the 4-day treatment can yield good outcome at another site than the originators’ place of work. Whether it works as well for other research groups and in other countries remains to be tested in future studies. However, the treatment is currently being tested in Iceland and an uncontrolled pilot study [28] obtained equally good effects as the original studies in Norway [3–5].

This approach has been developed as an integrated part of the specialist health care with severely affected patients and with virtually no bias in the selection of cases that are offered the treatment. Since clinical changes that are achieved during a very brief window of time are large, seen in a large majority of patients, and to a large extent are maintained at follow-up, we argue that the 4-day treatment might offer a unique longitudinal and nearly experimental approach to study basic mechanisms of brain plasticity. Because no other factors apart from treatment are of influence during the 4 days of intervention, it is an ideal setting for studies using functional magnetic resonance brain imaging (fMRI) or biomarkers of e.g. epigenetic changes to elucidate functional and structural changes on a number of different explanatory levels [29, 30]. This is an interesting area for future research which should be pursued.

## Conclusions

In sum, the Bergen 4-day treatment for OCD showed promising results. Future studies should test this treatment format using a RCT design.

## Abbreviations

DSM: Diagnostic and statistical manual of mental disorders
ERP: Exposure and response prevention
fMRI: Functional magnetic resonance imaging
GAD-7: Generalized Anxiety Disorder-7
LET: Lean into The anxiety
OCD: Obsessive-compulsive disorder
OUH: Oslo University Hospital
PHQ-9: Patient health questionnaire-9
SCID: Structured clinical interview
Y-BOCS: Yale-Brown obsessive-compulsive scale
